# Draft genome sequence of endophyte *Bacillus halotolerans* strain Can19L, isolated from the *Chenopodium album* L. leaves

**DOI:** 10.1128/mra.00540-25

**Published:** 2025-08-11

**Authors:** Vladimir K. Chebotar, Maria S. Gancheva, Margarita V. Khudyaeva, Oksana V. Keleinikova, Maria E. Baganova, Alexander N. Zaplatkin, Veronika N. Pishchik, Elena P. Chizhevskaya

**Affiliations:** 1 All- Russia Research Institute for Agricultural Microbiology, Pushkin, Russia; 2 Saint Petersburg State University, Saint Petersburg, Russia; The University of Arizona, Tucson, Arizona, USA

**Keywords:** draft Genome, endophyte, *Bacillus halotolerans*, Can19L, *Chenopodium album*, leaves

## Abstract

We report the genome sequence of the endophyte *Bacillus halotolerans* Can19L, isolated from the leaves of *Chenopodium album* L. The assembled genome consists of six contigs totaling 4,113,353 bp with a guanine–cytosine content of 43.7%.

## ANNOUNCEMENT

The endophytic strain Can19L was isolated from leaves of the salt-tolerant weed *Chenopodium album* L. collected in the Stavropol region, Russia (44°50'28.0"N 44°30'22.0"E), according to the protocol (1). Plant leaves were surface-sterilized, homogenized, and 100 µL aliquots of the resulting juice were plated on 1/20-diluted tryptic soy agar (Difco Laboratories, USA). Plates were incubated at 28°C for 3 days. A single colony was cultured in LB medium at 30°C overnight, and genomic DNA was extracted using the Wizard Genomic DNA Purification Kit (Promega, USA). DNA concentration was measured using a Qubit fluorometer, and 1000 ng of the extracted DNA was used to prepare a library with the KAPA HyperPlus kit (Roche, Switzerland). KAPA HyperPure beads (Roche, Switzerland) were used for a final purification and High Sensitivity DNA kit (Agilent Technologies, USA) was used for the library size distribution and quality assessment. Quantification of the library was performed using Quant-iT DNA Assay Kit (Thermo Fisher Scientific, USA). The resulting library was sequenced using the NextSeq 1000 platform (Illumina, USA), with the NextSeq 1000/2000 P1 Reagents (300 cycles) and 2% PhiX spike-in control. A total of 1,176,787 paired-end 150 bp reads were generated.

The quality of the raw reads was analyzed with FastQC v0.11.9 (2). The genome assembly was performed with SPAdes v3.14.1 (3) using the “careful” option. The FastANI v1.34 (4) tool was used to identify the closest reference genomes of the *Bacilli* class downloaded from the National Center for Biotechnology Information (NCBI) database (5). Can19L was found to be most closely related to *Bacillus halotolerans* (reference genome GCF_004006435.1) with an average nucleotide identity of 99.3%. Contigs were aligned to the reference genome for scaffolding using RagTag v2.1.0 (6), and then contigs shorter than 500 bp were removed using the reformat tool from BBMap (https://sourceforge.net/projects/bbmap/). PlasmidFinder-2.0 (7) was used to detect plasmids in the assembly but no plasmids were detected. Assembly metrics were evaluated using QUAST v5.1.0 (8), samtools coverage tool v1.13 (9), BUSCO v5.2.2 (bacillales_odb10) (10), and CheckM v1.2.3 (11). To calculate genome coverage, reads were mapped to the assembly using bwa (12). Unless otherwise noted, default parameters were used for all software. The assembled genome (85.7 × coverage) consists of six contigs totaling 4,113,353 bp, with an N50 value of 4,099,614 bp. The genome exhibits a GC content of 43.7% and shows high completeness (99.8%) with minimal contamination (0.59%).

The genome was annotated using the NCBI Prokaryotic Genome Annotation Pipeline v6.8 (13), which identified a total of 4,261 genes. Using antiSMASH v7.1.0 (14) with all extra features enabled, we identified gene clusters encoding biosynthesis pathways for fengycin, bacillaene, surfactin, bacilysin, subtilosin A, bacillibactin, laterocidine, and plipastatin. Previous studies demonstrated that *B. halotolerans* strain KKD1 enhances wheat growth and alleviates salinity stress by modulating phytohormone synthesis and osmoregulation (15). In this context, Can19L could be a valuable subject for future research.

## Data Availability

The genome sequence and raw reads were deposited in NCBI and can be accessed under BioProject PRJNA1180787 (SRA SRR31189800 and assembly GCA_044628115.1).
